# Conspicuity of prostate cancer on multiparametric magnetic resonance imaging: A cross‐disciplinary translational hypothesis

**DOI:** 10.1096/fj.202001466R

**Published:** 2020-09-13

**Authors:** Joseph M. Norris, Benjamin S. Simpson, Alex Freeman, Alex Kirkham, Hayley C. Whitaker, Mark Emberton

**Affiliations:** ^1^ UCL Division of Surgery & Interventional Science University College London London UK; ^2^ Department of Urology University College London Hospitals NHS Foundation Trust London UK; ^3^ Department of Pathology University College London Hospitals NHS Foundation Trust London UK; ^4^ Department of Radiology University College London Hospitals NHS Foundation Trust London UK

**Keywords:** conspicuity theory, multiparametric MRI, prostate cancer

## Abstract

Pre‐biopsy multiparametric magnetic resonance imaging (mpMRI) has transformed the risk stratification and diagnostic approach for suspected prostate cancer. The majority of clinically significant prostate cancers are visible on pre‐biopsy mpMRI, however, there are a subset of significant tumors that are not detected by mpMRI. The radiobiological mechanisms underpinning mpMRI‐visibility and invisibility of these cancers remain uncertain. Emerging evidence suggests that mpMRI‐visible tumors are enriched with molecular features associated with increased disease aggressivity and poor clinical prognosis, which is supported by short‐term endpoints, such as biochemical recurrence following surgery. Furthermore, at the histopathological level, mpMRI‐visible tumors appear to exhibit increased architectural and vascular density compared to mpMRI‐invisible disease. It seems probable that the genomic, pathological, radiological, and clinical features of mpMRI‐visible and mpMRI‐invisible prostate cancers are interrelated. Here, we propose a novel cross‐disciplinary theory that links genomic and molecular evidence with cellular and histopathological appearances, elucidating both the mpMRI visibility and clinical status of significant prostate cancer.

**Abbreviations**
68Ga: Gallium‐68
ADC: apparent diffusion coefficient
AUC: area under the curve
BCR: biochemical recurrence
BRCA: breast cancer gene
CCP: cell cycle progression
CD31: cluster of differentiation 31
CENPF: centromere protein F
CNA: copy number alterations
CT: computed tomography
DCE: dynamic contrast enhancement
DNA: deoxyribonucleic acid
DWI: diffusion‐weighted imaging
E2F: E2 transcription factor
ECM: extracellular matrix
GC: genomic classifier
GDF15: growth differentiation factor 15
GPS: genomic prostate score
HR: hazard ratio
Ki67: Kiel protein 67
mL: milliliter
mpMRI: multiparametric magnetic resonance imaging
MYC: myelocytomatosis oncogene
*n*
: number of patients
ng: nanogram
NICE: National Institute for Health and Care Excellence
OR: odds ratio
*p*
: probability
PET: positron emission tomography
PSA: prostate‐specific antigen
PSMA: prostate‐specific membrane antigen
PTEN: phosphatase and tensin homolog
Rad: RAD homolog, DNA repair protein
RB1: retinoblastoma gene 1
T2W: T2‐weighted imaging
tBCR: time to BCR
TRUS: transrectal ultrasound
TP53: tumor protein p53
UK: United Kingdom
VEGF: vascular endothelial growth factor
WNT‐b: Wingless and Int‐1 signaling pathway

## 1 INTRODUCTION

The advent of multiparametric magnetic resonance imaging (mpMRI) likely represents the single most important change to the diagnostic pathway for men with suspected prostate cancer in the past century. The traditional strategy of measuring serum prostate‐specific antigen (PSA) followed by systematic (semi‐random) transrectal ultrasound (TRUS)‐guided prostate biopsies is demonstrably poor as a diagnostic approach.^1^, ^2^ This results in an excess number of non‐image‐guided biopsies, and ultimately, under‐diagnosis of clinically significant cancer and overdiagnosis of clinically insignificant cancer.^2^ In contrast, pre‐biopsy mpMRI has excellent diagnostic accuracy for clinically significant prostate cancer, identifying the majority of important tumors, even before a biopsy is performed.^1^ The diagnostic accuracy afforded by mpMRI is now reflected by inclusion of the technology into national and international guidelines for men with suspected prostate cancer, including those produced by the National Institute for Health and Care Excellence (NICE) in the UK.^3^, ^4^

However, approximately 10%‐20% of clinically significant prostate cancers may appear invisible to mpMRI,^1^, ^5^, ^6^ producing understandable concern among clinicians and patients. The true clinical and oncological significance of mpMRI‐invisible disease remains uncertain. In contemporary urological practice, mpMRI is now often used as a first‐line triage tool to stratify those men that require a biopsy, and those that do not. If negative mpMRI is used as an indication to omit biopsy, then, there is a risk that men with mpMRI‐invisible cancer may harbor undiagnosed significant disease, that may result in unfavorable clinical outcome. As a result, there has been a recent growing research interest in characterizing the nature of disease that is both detected and undetected by mpMRI.^7^, ^8^

We propose a novel hypothesis that provides potential explanation for the conspicuity of prostate cancer on mpMRI, linking evidence from molecular, histopathological, radiological, and clinical research fields.

## 2 HYPOTHESIS: BRIDGING THE GAP BETWEEN BIOLOGY, PATHOLOGY, AND RADIOLOGY

Our hypothesis is that the positive mpMRI signal generated by visible prostate tumors is associated with multiple hallmarks of aggressive cancer, on a genetic, histopathological, and clinical level. Molecular evidence suggests that overexpression of proliferation‐regulating genes and loss of tumor suppressor genes leads to increased tumor growth, which is noted in aggressive prostate cancer, including cribriform pattern disease. We believe that histopathologically, these genetic features result in larger volume tumors, with increased tumor epithelial cell density, and thus, a reduced stromal to epithelial ratio. Rapid tumor growth stemming from these genetic influences results in areas of hypoxia, triggering vascularization signaling cascades, further increasing microvessel density, tissue density, and tumor size.

Radiologically, it is plausible that these features contribute to tumor visibility in each of the constituent MRI sequences that form part of the mpMRI assessment. First, increased tumor size is likely to directly result in increased lesion conspicuity, as the larger the tumor volume, the more likely it is to be above the spatial resolution limits of mpMRI.^9^ This is likely to be true for all of the mpMRI sequences, especially the anatomic T2‐weighted (T2W) sequence. Second, increased tumor tissue density manifests in restriction of movement of water molecules within malignant tissue, resulting in a stronger (more restricted) signal on the diffusion‐weighted imaging (DWI) sequence and apparent diffusion coefficient (ADC) map. Finally, increased vascular density may render tumors more apparent on mpMRI due to higher concentrations of contrast agent (in this case, gadolinium) accumulating in the additional vascular spaces, thus, generating stronger signal on the dynamic contrast‐enhanced (DCE) sequence.

Given these factors, it is plausible that larger, higher‐grade tumors, enriched with unfavorable genetic features, would be associated with poor prognosis. This then supports the notion that mpMRI‐visible cancer may confer a worse prognosis over mpMRI‐invisible counterparts.

Our integrated theory is consistent with the natural pathogenesis of cancer and with a number of studies which have investigated the clinical, histopathological, and genetic features of disease conspicuity on mpMRI, as we describe in detail here.

## 3 SUPPORTIVE EVIDENCE

The theory of prostate tumor visibility and invisibility on mpMRI should be addressed at three interconnected levels; the level of the organism, the level of tissue and cells, and the level of genetics.

### 3.1 Organism‐level: Clinical features of mpMRI‐visible disease

In cancer biology, organism‐level effects (ie, on patients with the disease) are ideally studied with long‐term clinical outcome data. However, large longitudinal, mpMRI‐correlated data sets are lacking to help clarify the association of clinical outcomes (such as, propensity for metastasis or prostate cancer‐specific mortality) with the mpMRI visibility status of prostate tumors. Despite this, evidence demonstrating the correlation of mpMRI visibility with shorter‐term endpoints does exist, and we can make reasonable inferences from this data. Furthermore, as will be discussed in subsequent sections, clinical risk of particular mpMRI phenotypes may also be estimated from adjunctive tests, including genetic biomarkers and the pathological assessment of tumor grade.

A commonly cited short‐term clinical endpoint in prostate cancer research is biochemical recurrence (BCR) following radical prostatectomy. Approximately 40% of patients with localized prostate cancer elect for radical prostatectomy as definitive therapy and a proportion of those that relapse after surgery go on to develop metastatic disease, eventually dying from prostate cancer.^10^, ^11^, ^12^, ^13^, ^14^, ^15^, ^16^ Therefore, it seems reasonable to assume a proportionality between BCR and longer‐term endpoints.

A recent study evaluated the ability of mpMRI to predict BCR within 3 years in men undergoing radical prostatectomy for prostate cancer (n = 91). The authors found that higher presurgery mpMRI scores (ie, tumors with greater mpMRI visibility) were associated with a greater likelihood of BCR with an overall area under the curve (AUC) of 0.64 (95% CI: 0.55‐0.73) indicating that tumor visibility on mpMRI has a good predictive ability to identify the most clinically aggressive tumors.^17^

Park and colleagues also evaluated the ability of tumor visibility on the combined sequences of mpMRI to predict BCR in a cohort of patients (n = 282) that underwent radical prostatectomy for prostate cancer.^18^ Using multivariate Cox regression, they showed that tumor visibility on combined mpMRI sequences independently predicted increased likelihood of BCR (*P* = .047) in similar manner illustrated by both PSA and Gleason score. Indeed, apparent tumor presence on mpMRI carried the greatest hazard ratio (HR: 2.38) for BCR compared to Gleason score at biopsy (HR: 1.34) and PSA (HR: 1.05).^18^ Similar results were demonstrated by Hattori et al (n = 314) who showed that patients undergoing radical prostatectomy for prostate cancer with mpMRI‐visible tumors carried the greatest risk for BCR (OR: 5.05, *P* = .031) in multivariate analysis compared to a Gleason score ≥ 8 at biopsy (OR: 4.85, *P* = .001) or a positive biopsy core ratio > 0.2 (OR: 3.19, *P* = .012).^19^ These results strongly suggest that conspicuity of cancer on mpMRI is an independent predictor of increased risk of disease recurrence following surgery, and as such, we might expect that a greater number of patients with mpMRI‐visible tumors would be at higher risk of prostate cancer‐specific mortality.

Obtaining reliable surrogate endpoints for mpMRI‐correlated clinical outcomes in a non‐prostatectomy setting remains complex, and studies investigating outcomes of mpMRI‐directed active surveillance are few in number. However, a recent study from our own institution demonstrated that mpMRI‐directed active surveillance (in which, re‐biopsy was omitted if there was no identifiable mpMRI‐visible lesion or radiological progression) showed that this imaging‐based approach had comparable outcomes to standard, non‐imaging‐based practice, and that mpMRI‐visible tumors that scored Gleason 3 + 4 had a reduced time to metastasis compared to similar mpMRI‐invisible tumors.^20^

### 3.2 Cellular‐level: Histopathological features of mpMRI‐visible disease

Through our recent systematic review of the pathological literature, we identified several interlinked histopathological hallmarks of tumor visibility on mpMRI.^21^ Compared to invisible disease, mpMRI‐visible tumors appear to have higher Gleason grading,^1^, ^22^ and increased tumor volume, cellular density,^23^, ^24^ microvessel density, and proportion of unusual aggressive subtypes (such as, cribriform^9^, ^25^ and intraductal carcinoma). Furthermore, it appears that cellular heterogeneity (eg, presence of acinar and foamy cell types) was found more frequently in mpMRI‐invisible disease compared to mpMRI‐visible disease, which tended to have more homogenous cell morphologies.^9^ Finally, prostate tumors arising in the transitional zone of the prostate also had reduced visibility on mpMRI compared to those originating in the peripheral zone, which had greater visibility.^9^

These histopathological properties are informative, as Gleason grade, tumor volume, tumor cellular density, microvessel density, presence of cribriform and intraductal carcinoma, and tumors arising in the peripheral zone are all features associated with increased likelihood of disease recurrence following surgery.^26^, ^27^, ^28^, ^29^, ^30^, ^31^ As such, these pathological observations further suggest that tumor mpMRI visibility may have genuine prognostic utility.

### 3.3 Genetic‐level: The genetic landscape of mpMRI‐visible disease

The genetic landscape that governs prostate tumor visibility on mpMRI is incompletely described. However, preliminary data are cohesive with our hypothesis and supports clinical and histopathological evidence, with enrichment of increased markers of disease aggressivity, including *PTEN* loss, *CENPF* expression, and higher genomic classifier scores, such as Oncotype and Decipher in mpMRI‐visible tumors compared to invisible disease. In our recent systematic review of the genetic literature, we identified three studies that had large accompanying genetic databases that were suitable for further bioinformatic analysis.^25^, ^32^, ^33^ Through this approach, we found that key genetic changes associated with extracellular matrix (ECM) components appeared to have differential enrichment in mpMRI‐visible disease, which may potentially explain several of the described pathological features (such as, increased cellular density) found in conspicuous tumors.

Summation of the extant literature reveals three major themes that describe the integration of molecular evidence with histopathological and clinical data, further supporting our cross‐disciplinary hypothesis. These themes are: hypoxia and angiogenesis, DNA damage and repair, and cell growth and proliferation.

#### 3.3.1 Hypoxia and angiogenesis

Establishing blood supply through induction of vascularization is a crucial hallmark of malignancy and is a likely determinant of tumor mpMRI visibility^34^ and as such, is a key part our hypothesis. Perhaps the most well‐known mediator of vascularization is *VEGF*, which encodes ligands that are involved in new blood vessel growth throughout embryonic and postnatal development.^35^ *VEGF* and microvessel density have been shown to associate with vascular perfusion in tumors on multiple imaging modalities, included computed tomography (CT), positron emission tomography (PET), and ultrasonography.^36^, ^37^ *VEGF* expression has been observed to be enriched in mpMRI‐visible prostate tumors and the response to anti‐*VEGF* therapy can be monitored using mpMRI.^38^, ^39^ *VEGF* expression and microvessel density additionally associates with tumor grade, metastasis and prognosis^40^ and this highlights a molecular component linking genetic, pathological, radiological, and clinical features of mpMRI‐visible prostate cancer. These features are also shared with hypoxia‐related signatures, which additionally appear to correlate with mpMRI‐derived imaging features.^41^

#### 3.3.2 DNA damage and repair

Tumor hypoxia is known to downregulate DNA double strand break repair gene expression in prostate cancer cells such as: *Rad51, Rad52, Rad54, BRCA1, BRCA2*, leading to an increase in DNA damage.^42^ We previously identified DNA damage as a key element of the genetic determinants of tumor visibility on mpMRI, from previous studies that demonstrated that tumors with higher DCE values (ie, tumors that are visible on the contrast mpMRI sequence) had significantly higher deregulation of DNA repair‐related genes, compared to tumors with lower DCE values.^43^ Li and colleagues also found that DNA damage repair pathway abnormalities were enriched in mpMRI‐visible tumors.^33^ Together, these studies provide support for our hypothesis, given that pronounced DNA repair abnormalities found in mpMRI‐visible disease are likely to contribute to an adverse clinical prognosis.

Recent evidence has revealed that the tumor suppressor gene, *PTEN*, is a critical regulator of the DNA damage response, specifically affecting double stranded break repair and nucleotide excision repair.^44^ *PTEN* loss has also been reported to be higher in mpMRI‐visible tumors across multiple studies,^45^, ^46^ further bolstering the concept of increased genetic risk in visible disease.

Finally, copy‐number burden has been directly correlated with the expression of hypoxia‐related genes and is known to correlate with poor prognosis.^47^ Kesch and colleagues found that copy number alterations (found in prostate tumors) were directly correlated to mpMRI and/or 68Ga‐PSMA‐PET/CT‐derived imaging features, with an increased frequency of losses in chromosomal loci, such as 1p, 2q, 6p, 6q, 11q, 12p, 15q, and 22q and, less frequently, gains in 3q.^48^ These results were confirmed by Houlahan and colleagues who found a greater copy‐number burden in mpMRI‐visible tumors in a cohort which utilized a matched Gleason grade and tumor volume methodology to account for the potentially confounding effects of adverse histopathology.^25^ To further increase the strength of association between genetic features, tumor visibility and clinical outcome, it appears that several of these regions (in particular, 3q) have been associated with aggressive clinical features in prostate cancer, again providing support for our proposed hypothesis.

#### 3.3.3 Cell growth and proliferation

**FIGURE 1:**
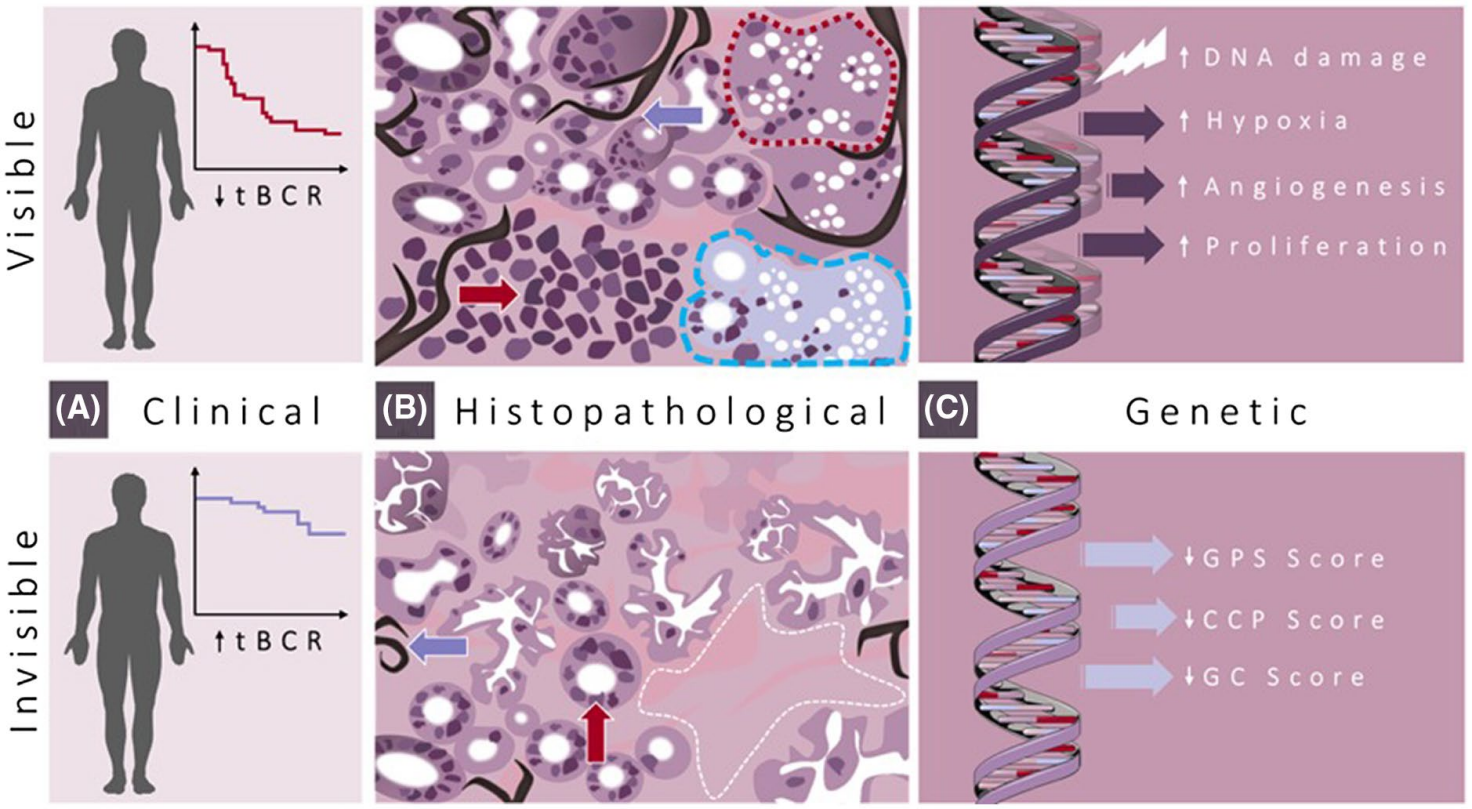
Integrated clinical, histopathological, and genetic aspects of mpMRI‐visible and mpMRI‐invisible prostate cancer. A, mpMRI‐visible disease appears to be associated with reduced time to biochemical recurrence (tBCR) following radical prostatectomy compared to increased tBCR in mpMRI‐invisible disease. B, Histopathological features of mpMRI‐visible disease include higher pathological grade, increased cell density (top red arrow), and increased microvessel density (top blue arrow) compared to lower cell density (bottom red arrow), microvessel density (bottom blue arrow), visible lesions have increased intraductal and cribriform architecture (dotted red line), and regions of hypoxia (dashed blue line) while mpMRI‐invisible tumors have increased areas of stroma (white dashed line). C, Genetic features of mpMRI‐visible disease include increased DNA damage and repair defects, increased hypoxic and angiogenic signaling, and increased proliferative signaling. mpMRI‐invisible disease is characterized by lower genomic prostate scores (GPS), cell‐cycle progression scores (CCP), and genomic classifier (GC) scores, indicative of lower risk malignancy

Perhaps the most relevant molecular characteristic that links to radiobiological mechanisms of tumor conspicuity is that mpMRI‐visible tumors appear to differentially express genes involved in cell growth and proliferation, when compared to mpMRI‐invisible tumors (Figure 1). Beksac and colleagues reported that pathways associated with cell cycle progression, specifically *PI3K‐AKT‐mTOR, E2F, MYC* target genes, and castration resistance genes (*WNT‐b*) tended to have the highest enrichment in the most visible tumors.^49^ Furthermore, Li and colleagues undertook an impressive multiphase project, showing that mpMRI‐visible tumors have increased expression of genes involved in mitotic cell cycle, protein folding, cell cycle, mitotic cell cycle process, and cell division. These included genes that encode proteins, such as *CENPF* and *GDF15*.^33^ As part of the in vivo phase of their investigation, they also showed that suppression of *CENPF* resulted in reduced mpMRI visibility of tumors and decreased cell growth rate, lowering cell density of tumors, decreasing the vascular marker *CD31* and proliferation marker *Ki67*.^33^ This increase in proliferative signaling in mpMRI‐visible cancer potentially explains previous observational reports that showed increased tumor size, cellular density, and microvasculature in conspicuous disease.^27^, ^28^, ^29^

## 4 CONFLICTING EVIDENCE

### 4.1 Organism‐level: Conflicting clinical evidence

There are a small number of studies that have found that higher‐grade tumors may be mpMRI‐invisible, however, as yet, these have not been linked to long‐term adverse clinical outcomes.^50^, ^51^, ^52^ Generally, these studies use pathological tumor grade to define clinical significance, and on the whole, lesion volume is not measured (or reported), which may suggest that the cited mpMRI‐invisible tumors are below limits of spatial resolution, and that they may become visible if they grew in size. Nonetheless, given the established close relationship between Gleason grade and clinical outcome,^53^ these studies do, at present, contradict our hypothesis.

### 4.2 Cellular‐level: Conflicting histopathological evidence

There are reports that certain aggressive prostate cancer subtypes, such as intraductal carcinoma and cribriform pattern disease, may have propensity for invisibility on mpMRI,^54^, ^55^, ^56^, ^57^ which potentially undermines our hypothesis outlined here. However, there are also reports that contradict this evidence, to some extent.^7^ Cribriform pattern cancer is closely associated with Gleason grade (specifically, Gleason grade 4) and is noted to have increased mitotic rate, vascular invasion, tissue necrosis,^58^ and association with distant metastasis and disease‐specific mortality,^58^, ^59^ which are all features suggestive of mpMRI‐visible disease.^1^, ^9^ Furthermore, tumor necrosis and low oxygenation levels (features found in cribriform cancer) are related to tumor size, which is another strong correlate of tumor visibility.^1^, ^60^ Overall, it seems that the small number of studies that report aggressive cribriform cancer to be an mpMRI‐invisible entity^56^ may be outweighed by a higher number of studies which demonstrate that most tumors containing this cancer subtype are visible on mpMRI.^9^, ^25^, ^61^

### 4.3 Molecular‐level: Conflicting genetic evidence

A proportion of the extant genetic evidence suggests that our hypothesis does not hold true for all tumors, with some mpMRI‐invisible cancers appearing to harbor potentially aggressive genes. However, there are studies that disagree with this assertion.^8^, ^21^ There are a small number of accounts that describe the genetic features of aggressive disease, such as copy number alterations in tumor suppressors *RB1* and *TP53*, as well as *MYC* amplification may be present in mpMRI‐invisible tumors.^62^ However, the potential effect of tumor suppressor mutations, in the context of an otherwise reassuring genetic environment on mpMRI‐invisible disease, is unknown. Furthermore, on the whole, these alterations are unlikely to be sufficient, in isolation, to cause truly aggressive disease, and are predominantly a feature of mpMRI‐visible tumors.^62^

## 5 THE CHALLENGE OF SIGNIFICANCE AND VISIBILITY THRESHOLDS

**FIGURE 2:**
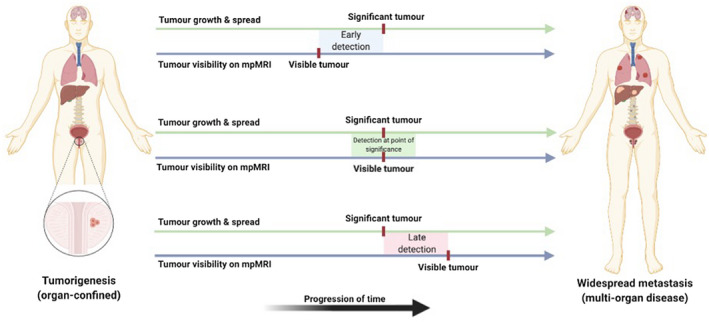
Potential scenarios for threshold alignment, between development of clinical significance (green line; from organ‐confinement through to metastases) and tumor visibility on mpMRI (blue line; from a small number of invisible cells to large visible tumors). Top: Tumor visibility may occur before onset of clinical significance, which risks overtreatment. Middle: Tumor visibility may occur at the point of development of clinical significance, which would represent an optimal treatment window. Bottom: Tumor visibility may occur after onset of clinical significance, which risks missing the window of curability

The concept of threshold management is intrinsic to the diagnosis and treatment of cancer and is important to consider when discussing our proposed hypothesis. Here, there are two thresholds to consider (Figure 2). First, the threshold for disease significance, in which there is a point on a theoretical spectrum (largely calibrated by tumor grade, size, and stage).^63^, ^64^ in which a cancer, having begun as a small number of malignant cells, grows, and obtains oncological potential to spread and impact both quality and quantity of life (ie, becomes clinically significant). Second, a threshold for disease visibility, in which there is a point on a theoretical spectrum where a cancer acquires sufficient characteristics (such as, size, vascularity, or density) to become visible on mpMRI.

It seems possible that tumor visibility on mpMRI may occur before onset of clinical significance, which carries the potential risk of disease overtreatment. Our hypothesis and the extant evidence suggest this is unlikely, and in fact, mpMRI appears to enable avoidance of detection of insignificant disease, overall.^1^, ^2^

Alternatively, tumor visibility may occur at the point of development of clinical significance, which would represent the best‐case scenario, providing diagnosis at the point at which treatment would be beneficial. This scenario supports our hypothesis, on multiple levels, however, the evidence used to construct this hypothesis is built on men with timely referral from primary care (eg, with PSA < 15 ng/mL), which may skew the mpMRI literature toward early detection.^1^

Finally, tumor visibility may occur after the point of clinical significance has past, in which the window for disease curability may be lost. In this instance, there are small number of research groups that have suggested that mpMRI may overlook a significant number of high‐grade prostate cancers,^65^ which clearly contradicts the hypothesis that we have described. However, these studies appear to be outliers, and key considerations of study methodology, population bias, definitions of significance, and quality of mpMRI acquisition and interpretation are likely to play a major role in their conclusions.

## 6 THE CHALLENGE OF FALSE POSITIVE MRI

Finally, an important observation in the field of prostate mpMRI is that not all mpMRI‐visible lesions transpire to be cancerous at biopsy.^1^ High suspicion for significant cancer on mpMRI (ie, with an mpMRI‐visible lesion) supports a higher likelihood of prostate cancer at biopsy,^1^ however, there remains a proportion of mpMRI‐visible lesions that do not prove to be cancerous on pathology. Indeed, depending on center‐experience and definitions of mpMRI visibility, almost 50% of suspicious lesions on mpMRI may transpire to be benign (or clinically insignificant disease) on biopsy.^2^

At present, “false positivity” on mpMRI is commonly attributed to a number of possibilities. First, there is the possibility of under‐sampling in which an mpMRI‐visible lesion represents a truly significant tumor, however, this is missed by the biopsy needle. Second, a positive mpMRI signal may be generated by benign features that recreate the radiological appearance of cancer. For instance, in prostatitis and inflammation, there is a dense collection of benign cells which may restrict movement of water (thus producing increased signal on the DWI sequence) or perhaps result in increased permeability of blood vessels (increased signal on DCE sequence). Third, there may be other mpMRI signal‐generating phenomena (eg, premalignant states) that have yet to be fully elucidated.

It seems possible that co‐occurring benign pathological features that are sufficient to generate positive mpMRI signal (eg, inflammation) may also play a role in prostate conspicuity on mpMRI. For instance, mpMRI‐visible cancers may have higher proportions of signal‐producing co‐occurring features compared to mpMRI‐invisible disease, however, this has yet to be investigated.

## 7 SUMMARY

The accumulation of recent evidence now creates a coherent scenario in which mpMRI‐visible prostate cancers have tendency to higher histopathological grade, larger tumor volume, increased cellular density, higher microvessel density, and lower ratio of stroma to epithelia (Table 1). The molecular evidence also matches this picture, with many genetic attributes seeming to align with these features. It seems biologically plausible that features such as cell density are driven by the genes regulating cell‐cell interactions, cell cycle, and proliferation, and that microvessel density may be governed by genes associated with angiogenesis and hypoxia‐inducibility. These features are also consistently associated with histopathological features of disease aggressivity, including higher Gleason grade and the presence of cribriform pattern disease. Our hypothesis, in which mpMRI‐visible tumors are likely to pose greater clinical risk than mpMRI‐invisible tumors, is supported by all of these elements.

**TABLE 1:** Summary of differences between mpMRI‐visible and mpMRI‐invisible prostate cancer

Feature type	Feature	mpMRI‐visible cancer	mpMRI‐invisible cancer	Refs
Clinical	Post‐prostatectomy BCR	Reduced time to BCR	Prolonged time to BCR	17, 18
	Time to metastasis	Reduced time to metastasis	Prolonged time to metastasis	20
Pathological	Gleason Grade	Higher overall & maximum grade	Lower overall & maximum grade	1, 22
	Tumor volume	Larger volume	Smaller volume	1, 22
	Cellular density	Higher cellular density	Lower cellular density	23, 24
	Microvessel density	Higher microvessel density	Lower microvessel density	23
	Stromal‐epithelial ratio	Lower stoma‐to‐epithelium ratio	Higher stroma‐to‐epithelium ratio	24
	Presence of cribriform	Increased presence of cribriform pattern	Reduced presence of cribriform pattern^a^	56, 61
	Presence of intraductal	Reduced presence of intraductal cancer	Increased presence of intraductal cancer	57
Genetic	Copy number variation	Increased copy number variation	Reduced copy number variation	25, 48
	DNA damage & repair	Higher deregulation of DNA repair genes	Lower deregulation of DNA repair genes	33, 43
	Biological pathways	Increased cell cycle, prolif., interaction	Reduced cell cycle, prolif., interaction	33, 49
	Angiogenesis	Increased angiogenesis promoters	Reduced angiogenesis promoters	33, 38
	Hypoxia	Increased hypoxia promoters	Reduced hypoxia promoters	42

Abbreviations: BCR, biochemical recurrence; DNA, deoxyribonucleic acid; mpMRI, multiparametric magnetic resonance imaging; prolif, proliferation; refs, references.

^a^ Some evidence suggests that cribriform pattern cancer has reduced mpMRI visibility.

As mpMRI becomes an increasingly intrinsic part of our diagnostic armamentarium for suspected prostate cancer, there is a growing need to understand why certain tumors evade detection. Here, we have expounded a hypothesis underlying the conspicuity of prostate cancer on mpMRI, drawing important links between evidence found at each possible level. Future phases of this research should focus on testing this theory, particularly with longitudinal mpMRI‐correlated data.

## CONFLICT OF INTEREST

Norris receives funding from the MRC. Simpson receives funding from the Rosetrees Trust. Whitaker receives funding from Prostate Cancer UK, the Urology Foundation and the Rosetrees Trust. Emberton, Freeman, and Kirkham have stock interest in Nuada Medical Ltd. Emberton receives funding from NIHR‐i4i, MRC, Sonacare Inc, Trod Medical, Cancer Vaccine Institute and Sophiris Biocorp for trials in prostate cancer. Emberton is a medical consultant to Sonacare Inc, Sophiris Biocorp, Steba Biotech, GSK, Exact Imaging and Profound Medical. Travel allowance was previously provided from Sanofi Aventis, Astellas, GSK, and Sonacare. Emberton is a proctor for HIFU with Sonacare Inc and is paid for training other surgeons in this procedure.

## AUTHOR CONTRIBUTIONS

All authors contributed equally to designing the research and writing the paper.
